# Noninvasive imaging of sialyltransferase activity in living cells by chemoselective recognition

**DOI:** 10.1038/srep10947

**Published:** 2015-06-05

**Authors:** Lei Bao, Lin Ding, Min Yang, Huangxian Ju

**Affiliations:** 1 State Key Laboratory of Analytical Chemistry for Life Science, School of Chemistry and Chemical Engineering, Nanjing University, Nanjing 210093, P.R. China; 2 Department of Pharmaceutical & Biological Chemistry, UCL School of Pharmacy, University College London, London WC1N 1AX, UK

## Abstract

To elucidate the biological and pathological functions of sialyltransferases (STs), intracellular ST activity evaluation is necessary. Focusing on the lack of noninvasive methods for obtaining the dynamic activity information, this work designs a sensing platform for *in situ* FRET imaging of intracellular ST activity and tracing of sialylation process. The system uses tetramethylrhodamine isothiocyanate labeled asialofetuin (TRITC-AF) as a ST substrate and fluorescein isothiocyanate labeled 3-aminophenylboronic acid (FITC-APBA) as the chemoselective recognition probe of sialylation product, both of which are encapsulated in a liposome vesicle for cellular delivery. The recognition of FITC-APBA to sialylated TRITC-AF leads to the FRET signal that is analyzed by FRET efficiency images. This strategy has been used to evaluate the correlation of ST activity with malignancy and cell surface sialylation, and the sialylation inhibition activity of inhibitors. This work provides a powerful noninvasive tool for glycan biosynthesis mechanism research, cancer diagnostics and drug development.

Glycosylation is an important posttranslational modification of proteins, and involves a set of functionally specific glycosyltransferases (GTs) located mainly in Golgi apparatus^1^. It plays critical roles in a variety of fundamental biological processes^2^, and appears to be particularly sensitive to malignant transformation^3^. GTs are therefore of significant interest as potential drug targets and tumor markers for diagnosis^1^,^4^. As one class of GTs, sialyltransferases (STs) that can introduce sialic acid (SA) to the terminal position of glycan chain attached on protein have attracted much considerable attention^5^. There are four families of STs in human body according to the carbohydrate linkages they synthesize: ST3Gal (α-2,3-ST), ST6Gal (α-2,6-ST), ST6GalNAc and ST8Sia (α-2,8-ST) families^6^. These STs are generally deregulated in cancer cells^3^. The increased Golgi-localized ST activity usually leads to more tumor associated carbohydrate antigens at cell surface. On most occasions, however, it has been noticed that the mechanisms of the altered sialylation in cancer cells and the correlation between the enzymatic activity and the appearance of sialylated glycans remain unknown^7^. To elucidate the biological and pathological functions of STs and use STs as diagnostic and therapeutic tools, sensitive methods for the assessment of ST activity have become urgent needs.

The common methods for ST assay involve the separation of STs from cellular lysate and the transfer of a radiolabeled sugar from the corresponding sugar-nucleotide in the presence of STs^8^. The complicated separation steps and handling of radioactive materials limit its application. Although the radioactive detection has been replaced with fluorescent labeling-based chromatography^9^ or fluorescence resonance energy transfer (FRET) analysis^10^, these separation-required methods cannot provide dynamic activity of STs in living cells, thus are difficult to verify the ST-related sialylation mechanisms.

To achieve the *in situ* noninvasive evaluation, a sensing system should be constructed to trace the sialylation process. Considering that STs transfer SA to a glycan chain, the method using genetically encoded peptides as substrate^11^ is not applicable to STs. Thus the sensing system should first deliver exogenous ST substrate into cells. To improve glycan recognition avidity and avoid cross-binding, a chemoselective and bioorthogonal probe can be integrated into the system to track the sialylation product in living cells. 3-Aminophenylboronic acid (APBA) has been known to form selectively stable complex with SA at physiological pH and even weak acid environment^12^,^13^, thus it is suitable for application in slightly acidic cellular organelles such as Golgi apparatus and other intracellular structures^14^. This work encapsulated tetramethylrhodamine isothiocyanate labeled asialofetuin (TRITC-AF) as ST substrate and fluorescein isothiocyanate labeled APBA (FITC-APBA) as the chemoselective recognition probe of sialylation product in a liposome-based delivery vesicle to design a novel sensing vesicle for *in situ* analysis of ST activity (Fig. 1). Different from glycoprotein fetuin containing 8.7% SA, the AF is a desialylated form of fetuin, thus was chosen as the substrate for ST^15^. FITC and TRITC could act as the donor and acceptor of a FRET pair, respectively^16^. After the vesicle was delivered into cells, the intracellular ST could transfer the SA from cytidine-5’-monophospho-sialic acid (CMP-SA), which exists in Golgi apparatus, to the terminal position of glycan chains of the TRITC-AF. Upon sialylation of the AF, the product could be recognized by the released FITC-APBA to bring FITC and TRITC close enough to achieve FRET. The signal intensity depended on the formation of the sialylated TRITC-AF, thus could be used for assessment of ST activity. This is the first report on the noninvasive analysis of ST activity in living cells.

## Results

### Preparation of TRITC-proteins and FITC-APBA

The TRITC-AF, TRITC labeled fetuin (TRITC-fetuin), TRITC labeled bovine serum albumin (TRITC-BSA) and TRITC labeled asialo-agalacto-fetuin (TRITC-AGF, AGF is the degalactosylated form of AF) were synthesized by linking isothiocyanate group to the amino group of proteins. AGF was acquired by removal of the exposed galactose residues from AF, thus both AGF and BSA could not be sialylated in the presence of ST, and were chosen as the negative control. FITC-APBA was prepared by binding the amino group of APBA to the isothiocyanate group of FITC (Supplementary Fig. S1).

### *In vitro* FRET between FITC-APBA and TRITC-proteins

**Figure 2:**
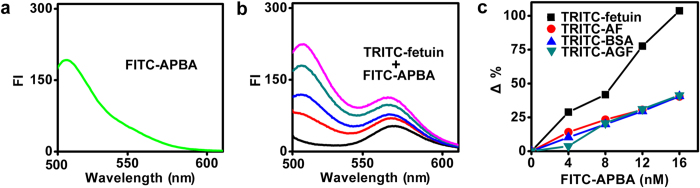
FRET responses of TRITC-proteins after incubation with FITC-APBA. (**a**) 12 nM FITC-APBA, and (**b**) TRITC-fetuin at a TRITC amount of 160 nM after incubation with 0, 4, 8, 12 and 16 nM FITC-APBA (from bottom to top) for 30 min under donor excitation. (**c**) Plots of FI change (Δ%) at 572 nm *vs* FITC-APBA concentration. Δ%=[*I*/*I*_C_ − 1]×100%, where *I* and *I*_C_ are the FI of TRITC-proteins in the presence and absence of FITC-APBA, respectively.

The feasibility of FRET from FITC-APBA to TRITC-fetuin was firstly validated *in vitro*. FITC-APBA and TRITC-fetuin showed maximum fluorescent emission at 519 and 572 nm, respectively (Fig.2 and Supplementary Fig. S2). After incubating the mixture of TRITC-fetuin and 12 nM FITC-APBA for 30 min, the fluorescent intensity (FI) of the mixture at 572 nm became 1.3 times larger than the sum of FI of TRITC-fetuin and FITC-APBA tested alone. The FI increased with the increasing concentration of FITC-APBA (Fig. 2b). As control, the mixture of FITC-APBA with TRITC-AF, TRITC-BSA or TRITC-AGF displayed higher FI of FITC at 519 nm and smaller FI change of TRITC at 572 nm at the same FITC-APBA concentration (Supplementary Fig. S2), indicating the quenching of FITC fluorescence and enhancement of TRITC fluorescence by efficient energy transfer from FITC to TRITC due to the binding of SA on fetuin with APBA. The fluorescence enhancement of TRITC-AF, TRITC-BSA or TRITC-AGF upon addition of FITC-APBA suggested the presence of relatively weaker FRET response (Fig. 2c), which was attributed to the adsorption of FITC-APBA on TRITC-proteins. The unspecific adsorption did not interfere with the dynamic analysis of the intracellular ST activity since it could be considered as a stable background of FRET and eliminated by software wizard. Considering the effect of FITC fluorescence on the FI measurement at 572 nm and the FRET response, an acceptor-to-donor concentration ratio of 13 was selected, at which the FRET response was sufficient for cellular imaging experiments.

### *In vitro* sialylation of TRITC-AF mediated by ST

**Figure 3:**
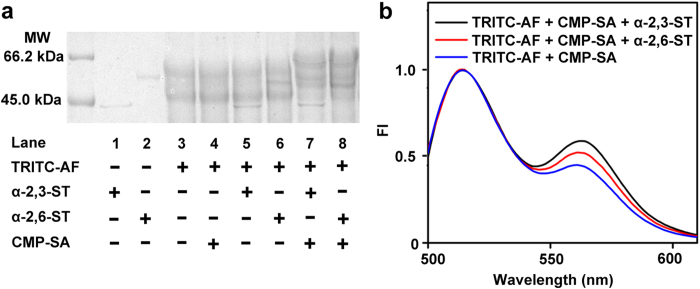
Confirmation of ST-mediated sialylation of TRITC-AF with electrophoresis and fluorescence assay. (**a**) SDS-PAGE of Tris-HCl buffer containing marked species after incubation at 37 ^o^C for 4 h. (**b**) Normalized fluorescent spectra of three mixtures after incubation at 37 ^o^C for 4 h and then reaction with FITC-APBA for 30 min, *λ*_ex_ = 488 nm.

The enzymatic activity of ST to transfer the SA from CMP-SA to TRITC-AF could be demonstrated using gel electrophoresis analysis and FITC-APBA mediated FRET assay. Only the coexistence of the three components could generate a new band with higher molecular weight than TRITC-AF, which corresponded to the sialylated product (Fig. 3a, lanes 7 and 8). After TRITC-AF was treated with α-2,3-ST from *Pasteurella multocida* or α-2,6-ST from *Photobacterium damsela* in the presence of CMP-SA and then reacted with FITC-APBA, the FI raised by 38.4% or 20.6% (Fig. 3b), indicating the formation of sialylated product of TRITC-AF, which led to the FRET response.

### Cellular delivery of TRITC-protein and FITC-APBA

**Figure 4:**
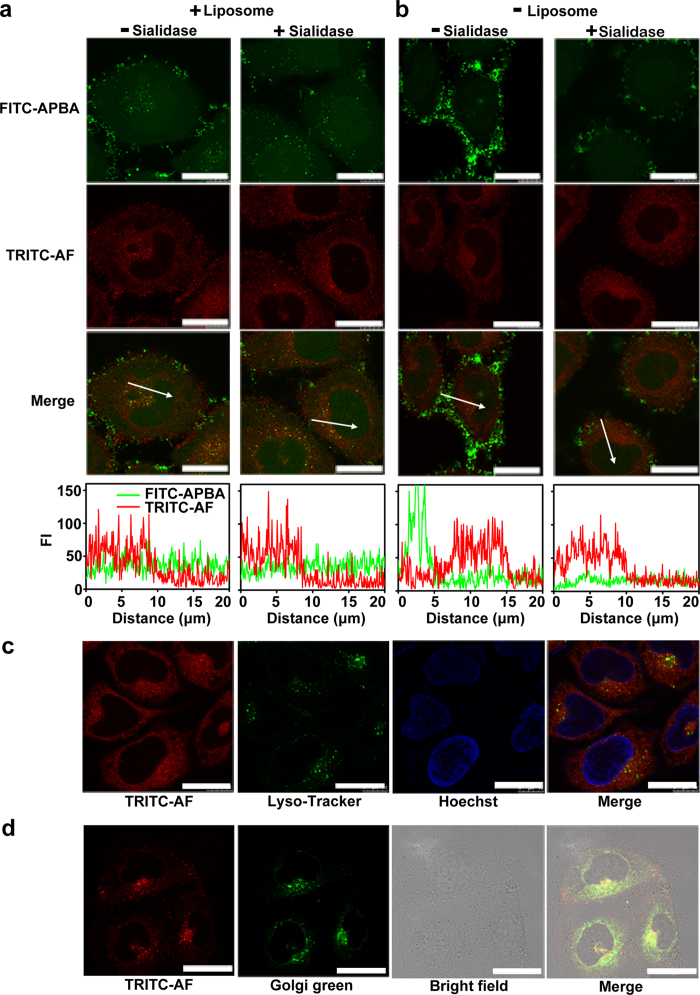
Delivery of TRITC-protein and FITC-APBA in the presence/absence of liposome into cells and localization of TRITC-protein in lysosome or Golgi apparatus. Confocal images of sialidase untreated and treated HeLa cells after delivery of 320 nM TRITC-AF and 12 nM FITC-APBA into cells in the presence (**a**) and absence (**b**) of liposome. FI of FITC-APBA and TRITC-AF were plotted as a function of distance along with white arrows. (**c**) Confocal images of HeLa cells incubated successively with liposome and 320 nM TRITC-AF for 120 min, Lyso-Tracker Green DND-26 for 30 min and Hoechst 33342 for 5 min. (**d**) Confocal and bright field images of HeLa cells incubated with liposome and 320 nM TRITC-AF for 120 min and then Golgi green (NBD-C6 ceramide) for 30 min. Scale bars, 20 μm.

By encapsulating FITC-APBA and TRITC-AF in Lipofectamine 2000 to form a sensing vesicle, the delivery of ST substrate and recognition probe into cells could be achieved with 120-min incubation. The vesicle-incubated human cervical carcinoma (HeLa) cells showed bright fluorescence spots of FITC and TRITC (Fig. 4a left). The fluorescence of FITC spread in whole cell area due to the release of FITC-APBA from the vesicles, while the fluorescence of TRITC-AF in perinuclear area was much stronger than that in nuclear area due to the much larger size of AF than APBA. Contrarily, in the absence of liposome, although the TRITC fluorescence could also be observed in perinuclear area, the incubated cells showed much brighter FITC fluorescence on the cell surface and very low FITC fluorescence in cytoplasm and nuclear area, which resulted from the strong binding of APBA to SA exposed on cell surface and SA-mediated weak uptake of FITC-APBA^17^,^18^ (Fig. 4b left). The two probes failed to generate FRET in the absence of liposome as a result of the deficiency of FITC-APBA in cytoplasm (Supplementary Fig. S3). To verify the function of SA in cellular delivery, sialidase was used to cleave the SA from cell surface. After cleaving SA, the vesicle-incubated HeLa cells showed the same fluorescence signals as those without SA cleavage (Fig. 4a right), while sialidase and then FITC-APBA/TRITC-AF treated cells showed obviously different fluorescence distribution from those without sialidase treatment (Fig. 4b right). The cleavage of SA from cell surface greatly decreased fluorescent signals both on cell surface and in the cell area. Thus in the presence of liposome the cellular delivery was unrelated to cell surface SA, which excluded the effect of cell surface SA on the detection of ST activity.

Although the fluorescence of TRITC-AF in the absence of liposome could also be observed in cytoplasm of sialidase-treated cells owing to the protein receptor mediated endocytosis, the liposome-assisted TRITC-AF delivery ensured the escaping of the vesicle from endosome/lysosome to cytosol^19^ and its entering into Golgi complex^20^. The former could be demonstrated by staining lysosomes and nucleus of TRITC-AF/liposome incubated HeLa cells with Lyso-Tracker Green DND-26 and Hoechst 33342, respectively. The confocal images indicated the successful escape of TRITC-AF from endosome/lysosomes^21^, thus it distributed throughout the cytoplasm (Fig. 4c). The staining experiment with Golgi green verified the entering of TRITC-AF in Golgi complex (Fig. 4d), where the presence of ST and CMP-SA ensured the sialylation of TRITC-AF.

### Confirmation of intracellular FRET signal

**Figure 5:**
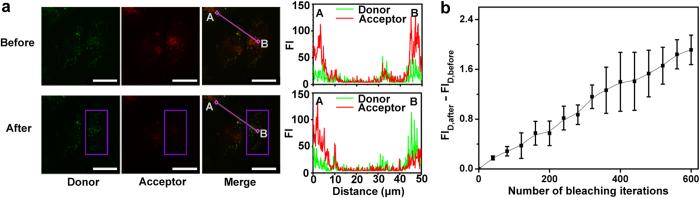
Confirmation of intracellular FRET. Confocal images of HeLa cells incubated with FITC-APBA and TRITC-fetuin encapsulated liposome before and after acceptor photobleaching at marked rectangle area for 600 times (**a**, left), and linear FI scans of donor and acceptor from point A to point B (**a**, right). Scale bars, 20 μm. (**b**) FI change of donor during acceptor photobleaching. Data and error bars show the average and S.D. of three replicates.

Acceptor photobleaching experiment was carried out to address the intracellular FRET. After acceptor photobleaching, the enhanced emission from FITC was observed in the dequenching area of HeLa cells incubated with liposome containing FITC-APBA and TRITC-fetuin (Fig. 5a, After, point B and Fig. 5b), indicating the recovery of FITC due to the photobleaching of TRITC and the FRET from FITC to TRITC before the photobleaching.

### Assessment of ST activity in living cells

**Figure 6:**
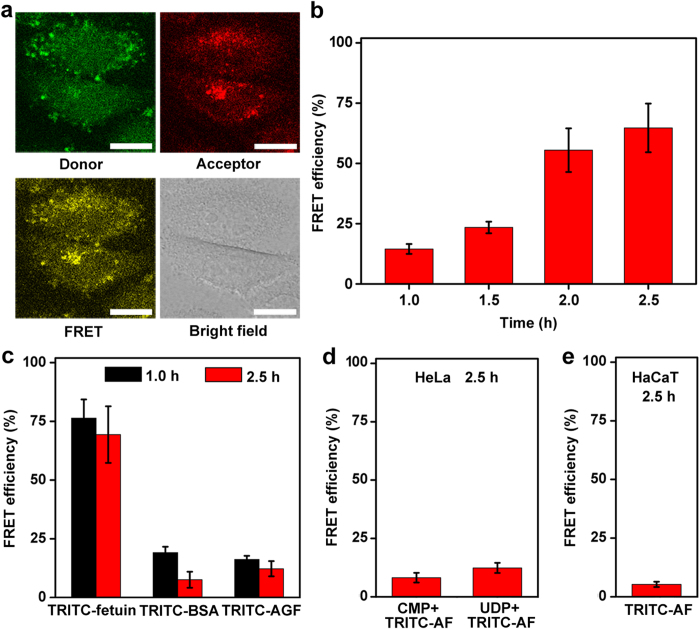
Assessment of intracellular ST activity of living cells using TRITC-protein. (**a**) Confocal and bright field images of G1-phase HeLa cells after transfected with sensing vesicle for 2.0 h and then cultured in growth medium for 1.0 h. Scale bars, 20 μm. (**b**) Time-course of FRET efficiency averaged across cytoplasm region during sialylation of TRITC-AF in G1-phase HeLa cells. (**c**) FRET efficiency in G1-phase HeLa cells after incubated with liposome vesicles containing FITC-APBA and TRITC-proteins for 2.0 h and then cultured in growth medium for 1.0 and 2.5 h. (**d**) and (**e**) FRET efficiency in G1-phase HeLa cells treated with CMP and UDP as inhibitors and normal G1-phase HaCaT cells after incubation with sensing vesicle. Data and error bars show the average and S.D. of three replicates.

The liposome-based sensing system was firstly applied for dynamically tracing the product of ST-mediated sialylation. Considering the fact that the ST activity is related to cell proliferation and cell cycle, especially G1 phase^22^,^23^, the HeLa cells used for ST activity analysis were synchronized at the beginning time of G1 phase (Supplementary Fig. S4). After G1-phase HeLa cells were transfected with sensing vesicle for 2.0 h and then incubated in growth medium for 1.0 h, the confocal image showed obvious FRET signal (Fig. 6a). The time-course images of FRET efficiency indicated the increase of sialylated product in cytoplasm of G1-phase HeLa cells (Supplementary Fig. S5). The FRET efficiency extracted across cytoplasm region increased from 14.5% to 64.7% with a statistically significant change (p < 0.05) during incubation period from 1.0 to 2.5 h (Fig. 6b). This result demonstrated the feasibility of the proposed strategy for noninvasive tracing of the ST-mediated sialylation process.

The specificity of TRITC-AF sialylation in Golgi complex of living cells was demonstrated with TRITC-fetuin, TRITC-BSA and TRITC-AGF. After transfecting the G1-phase Hela cells with the vesicle containing these proteins and FITC-APBA, the FRET efficiency decreased with the increasing incubation time in growth medium (Fig. 6c and Supplementary Fig. S6a), which was opposed to that in TRITC-AF sialylation process (Fig. 6b). The FRET efficiency for TRITC-fetuin kept at a high level in the observed period (76.4% to 69.4%) due to the conjugation of FITC-APBA and SA exposed on TRITC-fetuin. However, TRITC-BSA or TRITC-AGF showed a very low FRET efficiency, which resulted from the adsorption of FITC-APBA on these proteins. The decrease in FRET efficiency could be attributed to the desorption of non-covalent linked FITC-APBA from TRITC-proteins. At 2.5-h incubation, the FRET efficiency of 64.7% for TRITC-AF was close to that for TRITC-fetuin (Fig. 6b,c), indicating the formation of sialylated TRITC-AF in living cells in the presence of intracellular ST. The sialylation process in S-phase HeLa cells was also studied (Supplementary Fig. S6b). Different from that of G1-phase cells, after transfected with the vesicle, the FRET efficiency of the S-phase HeLa cells slightly decreased from 1 to 1.5 h, indicating the lack of ST activity in S-phase HeLa cells.

The proposed strategy was further employed for monitoring the ST activity of G1-phase HeLa cells during drug treatment. CMP and uridine-5’-diphosphate (UDP), the inhibitors of ST activity^24^ and SA biosynthesis^25^, were chosen as model drugs, respectively. After drug treatment, these cells displayed much lower FRET efficiency (8.2% and 12.4%, respectively) (Fig. 6d and Supplementary Fig. S6c) than those without treatment at 2.5-h incubation in growth medium (Fig. 6b), just like the negative controls (Fig. 6c), indicating the effective inhibition of CMP to the ST activity and the influence of UDP on SA biosynthesis.

The proposed platform could be used to discriminate tumor and normal cells by monitoring the intracellular ST activity. After transfection with the sensing vesicle the G1-phase normal human skin keratinocytes (HaCaT cells) showed a FRET efficiency of 5.3% (Fig. 6e and Supplementary Fig. S6c), indicating low expression of ST level in normal cells. Similarly, the SA expressed on the surface of HeLa cells was also 5.7-fold more than that on HaCaT cells (Supplementary Fig. S7), indicating the correlation between intracellular ST activity and the expression of surface sialylated glycans.

## Discussion

In this study, we design a noninvasive sensing system to trace sialylation product and detect intracellular ST activity using FRET technique. By virtue of encapsulation in liposome, TRITC-AF and FITC-APBA are delivered into cells and released from endosome/lysosomes, then well dispersed in cytoplasm. AF is chosen as the glycoprotein substrate of STs, because it contains well-characterized *N*-linked and *O*-linked oligosaccharides that are recognized as the acceptor substrates by nearly all kinds of STs^26^,^27^,^28^. After some of the TRITC-AF enter the Golgi complex, they are sialylated by ST. Since the sialylation is the last step of glycan chain terminal modification, the SA-modified TRITC-AF can be transported from Golgi complex into cytoplasm and finally displayed on cell surface or secreted into extracellular matrix^29^. Meanwhile, the APBA moieties of FITC-APBA dispersed in cytoplasm are able to bind with SA in a chemoselective way by forming a remarkably stable trigonal structure in slightly acidic cellular organelles^14^,^30^. As a result, the newly sialylated TRITC-AF can be discriminated by the FRET signal from FITC to TRITC. Moreover, to exclude the energy transfer-irrelevant FITC and TRITC emissions excited at the donor excitation wavelength, FRET efficiency is extracted for intracellular ST activity assay, which is calculated automatically by Leica software based on sensitized acceptor emission rather than the distance between the two fluorophores^31^. The bright dots in the FRET efficiency images (Supplementary Figs S5 and S6) show the location of sialylated product, with intensity level reflecting the ST activity (higher value corresponds to greater ST activity).

The obtained FRET efficiency signal using TRITC-AF as the substrate indicates the activity of three types of STs, including ST3Gal (α-2,3-ST), ST6Gal (α-2,6-ST) and ST6GalNAc, toward glycoproteins in an individual cell, thus reflecting the overall ST activities in normal physiological environment. This is different from other ST activity assessment strategies performed *in vitro*^9^,^10^. As a result, this sensing system can be exploited to assess the total ST activity level toward glycoprotein of a living cell and investigate the ST-related biological process under complex cellular regulation. Although the endogenous ST substrates can also undergo the sialylation process along with the exogenous TRITC-AF, they do not affect the dynamic evaluation of ST activity.

During monitoring of the ST-mediated sialylation process, significant increase of the FRET efficiency has been observed for G1-phase HeLa cells (Fig. 6b). Due to the fact that sialoglycoconjugates (including sialoglycoproteins and sialogangliosides) play important roles in cell cycle and apoptosis, such as cell cycle reactivation^32^ and modification^33^,^34^,^35^, the synchronized cells are used for ST activity tracking experiment. During G1 phase, glycoconjugate sialylation is necessary for C6 glioma transit through G1^22^ and astrocytic proliferation^23^, and the ST activity is found to be upregulated in C6 glioma^22^ and human B lymphocytes^36^. As comparison this work investigates the ST activity in S-phase HeLa cells, which show negligible variation during the 1.5-h observation time (Supplementary Fig. S6b). The greater ST activity in G1-phase than S-phase HeLa cells is the first time report. In addition, considering that an emerging aspect of anticancer therapy is the inhibition of ST^37^, this work further demonstrates that the system can be conveniently applied to monitoring of the ST activity of G1-phase HeLa cells during drug treatment, thus providing an efficient platform for development and screening of ST-related anticancer drugs.

Aberrant overexpression of sialylation is the hallmark of cancer progression. A positive correlation has been found to exist between the cell surface sialylation level and glycoenzymes belonging to different classes^3^,^38^. For example, the cancer-related alteration in ST expression is often regarded as the basis of deranged expression of sialylated structures^6^. Considering the fact that the expression of cell surface SA depends on the status of all SA-modulatory enzymes, including primarily STs and sialidases, which just have opposite effect^39^, the ST activity of living cells might be masked when SA analysis is only performed on the cell surface. The proposed strategy has revealed higher ST activity both in cancer cells and on their surface compared with normal cells (Fig. 6b,e and Supplementary Fig. S7), which is consistent with the reports, demonstrating the feasibility of the method as a powerful tool for uncovering the glycan-involved cellular functional mechanisms behind the phenomena.

In conclusion, a sensing system for noninvasive FRET assessment of intracellular ST activity and tracing of sialylation product in living cells has been developed by encapsulating TRITC labeled exogenous ST substrate and FITC labeled chemoselective recognition probe of sialylation product in liposome-based delivery vesicle. Through FRET efficiency analysis, the proposed system can be used to probe the correlation of intracellular ST activity with malignancy and cell surface SA expression, the intracellular sialylation process and the inhibition of inhibitor to the sialylation. This strategy can be facilely adapted to design other sensing systems for intracellular GT analysis. It offers a valuable tool for clinical diagnostics, drug development, understanding of glycan biosynthesis mechanism, and study of GT-mediated biological process.

## Methods

General experimental methods, reagents, cell culture and synchronization, flow cytometric analysis of synchronized HeLa cells are described in the Supplementary Information.

### Preparation of AGF

AGF was prepared by treating AF with β-galactosidase from *Escherichia coli*. 10 mg AF was dissolved in 2 mL of 50 mM Tris-HCl buffer (pH 7.2) containing 10 mM magnesium chloride, 5 mM β-mercaptoethanol and 200 U β-galactosidase. The reaction was allowed to proceed for 48 h at 37 ^o^C, and then AGF (MW 42 kDa) was separated from β-galactosidase (MW 540 kDa, four equal subunits of 135 kDa) by ultrafiltration (Amicon Ultra-0.5, 100 k MWCO, Millipore) and purified by ultrafiltration (Amicon Ultra-0.5, 30 k MWCO, Millipore) against ultrapure water.

### Preparation of TRITC-proteins

TRITC (1 mg mL^−1^) in anhydrous DMSO was prepared freshly. 3 mg AF, fetuin, BSA and AGF were dissolved in 1 mL 100 mM carbonate/bicarbonate buffer (pH 9.0), respectively. While stirring, 35 μL of TRITC was slowly added to the protein solution. The mixture was incubated over night at room temperature in the dark. Ultrafiltration (Amicon Ultra-0.5, 30 k MWCO, Millipore) was used to separate and concentrate the TRITC-proteins at 8000 rpm for 10 min. The obtained conjugates were washed more than 6 times with PBS to remove excess and hydrolyzed TRITC. The molar ratios of TRITC to proteins were obtained by determining the absorbance of TRITC at 555 nm with a molar extinction coefficient of 85000 M^−1^ cm^−1^ and the absorbance of AF, fetuin, BSA and AGF at 280 nm with the percent extinction coefficients of 4.9, 4.5, 6.67 and 5.19, respectively. Due to the effect of TRITC absorbance at 280 nm, a correction factor of 0.34, which was obtained from the absorbance of TRITC at 280 and 555 nm, was used for determination of protein concentrations. The molar ratios of TRITC to proteins were characterized to be 0.5, 0.3, 1.0 and 0.3, respectively.

### Synthesis of FITC-APBA

FITC-APBA was prepared by a modified procedure^40^. APBA (29 mg, 0.21 mmol) and FITC (80 mg, 0.21 mmol) were firstly dissolved in methanol (50 mL). After reaction for 24 h at 30 ^o^C under stirring and free of light, the resulting mixture was evaporated under reduced pressure. The residue was then purified by silica gel column chromatography (EtOAc/EtOH 5:1) and evaporated under reduced pressure to obtain FITC-APBA as an orange-yellow powder (49.6 mg, 46% yield) (Supplementary Fig. S1). ^1^H NMR (500 MHz, DMSO-d6): δ 10.15 – 10.10(m, 2H), 10.04 (s, 1H), 8.17 (d, *J* = 1.6 Hz, 1H), 8.06 (d, *J *= 19.2 Hz, 2H), 7.81 (dd, *J *= 8.3, 1.9 Hz, 1H), 7.74 (s, 1H), 7.62 (d, *J *= 7.3 Hz, 1H), 7.54 (d, *J *= 8.4 Hz, 1H), 7.34 (t, *J *= 7.7 Hz, 1H), 7.20 (d, *J *= 8.3 Hz, 1H), 6.68 (d, *J *= 1.9 Hz, 2H), 6.63 – 6.56 (m, 3H); ^13^C NMR (126 MHz, DMSO-d6): δ 179.85, 168.45, 159.47, 151.87, 147.68, 141.31, 138.27, 134.88, 130.91, 130.58, 129.99, 129.02, 127.69, 126.43, 126.28, 123.89, 117.76, 112.59, 109.67, 102.24, 83.04; HRMS (*m*/*z*): [M+H]^+^ calcd. for C_27_H_20_BN_2_O_7_S, 527.3329; found, 527.1593.

### *In vitro* FRET between FITC-APBA and TRITC-proteins

TRITC-protein solutions (20 μL, containing 1.6 μM TRITC) were mixed with 0, 5, 10, 15 and 20 μL FITC-APBA solutions (0.16 μM) and diluted to 200 μL with PBS, respectively. These mixtures were incubated for 30 min at room temperature and subjected to fluorescence measurements with an excitation of 488 nm for FITC as donor and an emission of 572 nm for TRITC as acceptor. Although the FITC-APBA displayed a higher donor peak compared to the acceptor peak, which was consistent with reported phenomena^41^,^42^, it would not interfere with the extraction of FRET efficiency.

### Substrate tolerance of ST toward TRITC-AF

The enzymatic activity of α-2,3-ST and α-2,6-ST toward TRITC-AF was verified through *in vitro* sialylation experiment. After TRITC-AF (3.2 μM), CMP-SA (100 μM) and ST (20 mU mL^−1^) were added in Tris-HCl buffer (100 mM, pH 8.0) containing MgCl_2_ (5 mM), MnCl_2_ (5 mM) and 0.1% Triton-X 100 to a total volume of 200 μL and subjected to reaction for 4 h at 37 ^o^C, the enzymatic product was separated and concentrated by ultrafiltration with Tris-HCl, and then loaded onto a sodium dodecyl sulfate polyacrylamide gel. After electrophoresis, the gel was washed with water and then stained with coomassie blue. The enzymatic product was also separated and concentrated by ultrafiltration with PBS, and then mixed with FITC-APBA (15 μL, 1.6 μM in PBS) to a total volume of 200 μL, which was incubated for 30 min and then purified by ultrafiltration to remove excess FITC-APBA. The conjugate was diluted to 200 μL with PBS for fluorescence detection with an excitation of 488 nm for FITC and an emission of 572 nm for TRITC.

### Delivery of TRITC-protein and FITC-APBA into cells

Liposomal vesicle transfection (Lipofectamine 2000, Invitrogen) was used to introduce TRITC-protein and FITC-APBA into cells. 1 μL FITC-APBA (1.2 μM in PBS) and 1 μL TRITC-protein (16 μM TRITC in PBS) were added in 20 μL Opti-MEM I reduced serum medium (Opti-MEM, Invitrogen). 0.5 μL lipofectamine 2000 was dissolved in 20 μL Opti-MEM and incubated for 5 min. After two solutions were mixed and incubated for 20 min, the liposomal vesicle was formed, which was then added into a cell-adhered dish containing 60 μL Opti-MEM to incubate for 2.0 h. Afterward the medium was replaced with DMEM containing 10% FCS as growth medium and incubated for 1.0 h to remove the excessive vesicle. The transfected cells were finally observed on laser scanning confocal microscope (Leica TCS SP5). The FITC-APBA emission in the range of 500-530 nm was excited at 488 nm, and the TRITC-protein emission in the range of 560-620 nm was excited at 543 nm.

The SA-cleaved HeLa cells was used to verify the function of SA in cellular delivery and obtained by culturing the cells in a DMEM medium containing 100 mU mL^−1^ sialidase for 60 min at 37 °C followed by washing twice with PBS.

### Subcellular localization

After the cells transfected with TRITC-AF/liposome were rinsed with DMEM, Lyso-Tracker Green DND-26 (Invitrogen) was added to the cell-adhered chamber with a final concentration of 75 nM to incubate for 30 min, which was then rinsed with medium and treated with 10 μg mL^−1^ Hoechst 33342 (Sigma-Aldrich) in PBS for 5 min. Finally, the living cells were rinsed and imaged in DMEM. 405 and 488 nm lasers were used for Hoechst 33342 and Lyso-Tracker Green DND-26 excitation and the fluorescence signals in the emission range of 460-490 nm and 500-530 nm were collected, respectively.

To analyze the Golgi apparatus, 10 μM Golgi Green (NBD-C6 ceramide) (KeyGen Biotech) diluted in Hank’s buffered salt solution (HBSS) (KeyGen Biotech) was added to the cell-adhered chamber to incubate for 30 min at 4 °C in the dark. Subsequently, the cells were incubated with HBSS for 30 min at room temperature in the dark. The living cells were then rinsed and imaged in DMEM. 458 nm laser was used for Golgi Green (NBD-C6 ceramide) excitation and the fluorescence signal in the emission range of 510-540 nm was collected.

### Confirmation of intracellular FRET signal

Acceptor photobleaching module (FRET AB Wizard) of the Leica software was used for donor dequenching experiments taken on a confocal microscope. A selected region of the cells transfected with liposome containing FITC-APBA/TRITC-fetuin was exposed to 543 nm laser (the excitation wavelength of TRITC) at high-power (80%) to photobleach the acceptor. One image was captured for each bleaching process. The fluorescence signals of donor before and after acceptor photobleaching were collected in the emission range of 500-530 nm with an excitation of 488 nm.

### Intracellular ST activity assay by FRET imaging

Sensitized emission module (FRET SE Wizard) of the Leica software was used for transfected cell FRET analysis on a confocal microscope. The FRET signal was collected in the emission range of 560-620 nm with an excitation of 488 nm. FRET efficiency was used to quantify the activity of intracellular STs in order to minimize the effect of stable background. The data for the calculation included the FRET signal, the donor emission in the range of 500-530 nm, which was excited at 488 nm, and the acceptor emission in the range of 560-620 nm, which was excited at 543 nm.

### Sialylation inhibition experiments

The G1-phase HeLa cells treated with drugs were acquired by culturing HeLa cells at the beginning time of S phase in growth medium containing 3 mM CMP or UDP for 12 h. Then the cells were maintained in Opti-MEM containing 3 mM CMP or UDP and incubated with sensing vesicle formed in the same medium for 2.0 h. Afterward the medium was changed to growth medium and incubated for 1.0 h before intracellular ST activity assay.

### Detection of cell surface SA expression

HeLa cells (0.5 mL, 1 × 10^6^ mL^−1^) and HaCaT cells (0.5 mL, 1 × 10^6^ mL^−1^) were cultured in a 20-mm dish and synchronized at the beginning time of G1 phase, respectively. After the HeLa and HaCaT cells were incubated with FITC-APBA (0.5 mL, 20 nM in PBS) for 10 min and rinsed with PBS, confocal imaging was used to demonstrate the cell surface SA expression extent.

## Additional Information

**How to cite this article**: Bao, L. *et al.* Noninvasive imaging of sialyltransferase activity in living cells by chemoselective recognition. *Sci. Rep.* **5**, 10947; doi: 10.1038/srep10947 (2015).

## Supplementary Material

Supplementary Information

## Figures and Tables

**Figure 1:**
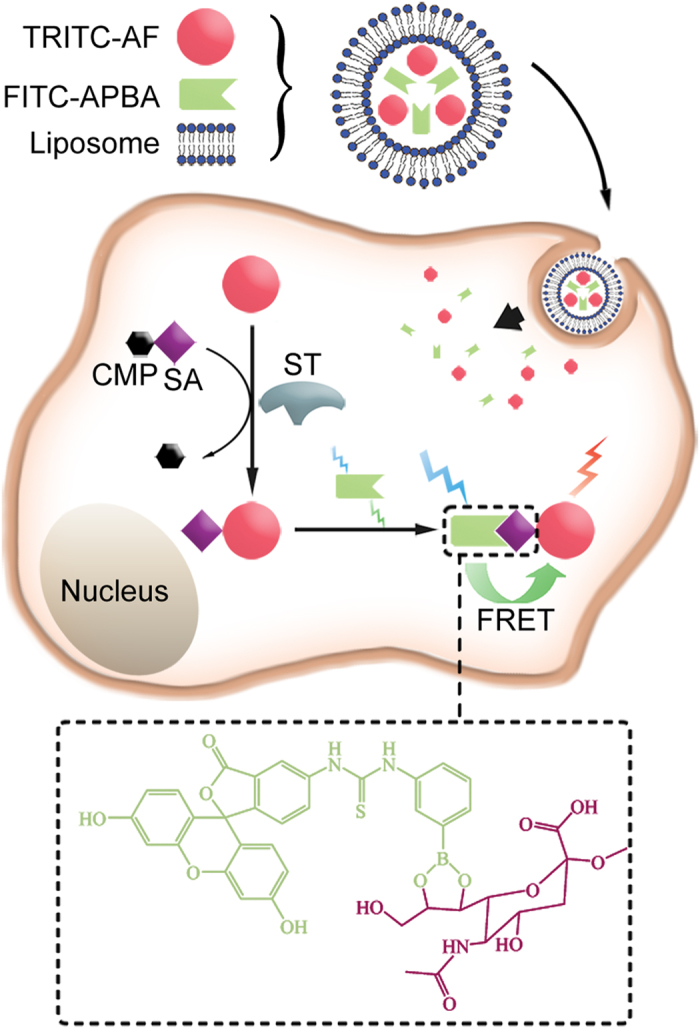
FRET-based sensing system for noninvasive imaging of intracellular ST activity. Schematic illustration of sensing vesicle, FRET sensing principle and chemical structure of the FITC-APBA/SA complex.

## References

[b1] Fuster M. M. & Esko J. D. The sweet and sour of cancer: glycans as novel therapeutic targets. Nat. Rev. Cancer 5, 526–542 (2005). 16069816 10.1038/nrc1649

[b2] Rabinovich G. A. & Toscano M. A. Turning ‘sweet’ on immunity: galectin-glycan interactions in immune tolerance and inflammation. Nat. Rev. Immunol. 9, 338–352 (2009). 19365409 10.1038/nri2536

[b3] Drake P. M. *et al.* Sweetening the pot: adding glycosylation to the biomarker discovery equation. Clin. Chem. 56, 223–236 (2010). 19959616 10.1373/clinchem.2009.136333 PMC2849286

[b4] Dube D. H. & Bertozzi C. R. Glycans in cancer and inflammation-potential for therapeutics and diagnostics. Nat. Rev. Drug Discov. 4, 477–488 (2005). 15931257 10.1038/nrd1751

[b5] Bos P. D. *et al.* Genes that mediate breast cancer metastasis to the brain. Nature 459, 1005–1009 (2009). 19421193 10.1038/nature08021 PMC2698953

[b6] Dall’Olio F. & Chiricolo M. Sialyltransferases in cancer. Glycoconj. J. 18, 841–850 (2001). 12820717 10.1023/a:1022288022969

[b7] Harduin-Lepers A. *et al.* Sialyltransferases functions in cancers. Front Biosci (Elite Ed) 4E, 499–515 (2012). 10.2741/e396 22201891

[b8] Grimes W. J. Sialic acid transferases and sialic acid levels in normal and transformed cells. Biochemistry 9, 5083–5092 (1970). 4320586 10.1021/bi00828a007

[b9] Yu H. *et al.* A multifunctional pasteurella multocida sialyltransferase: a powerful tool for the synthesis of sialoside libraries. J. Am. Chem. Soc. 127, 17618–17619 (2005). 16351087 10.1021/ja0561690

[b10] Washiya K., Furuike T., Nakajima F., Lee Y. C. & Nishimura S.-I. Design of fluorogenic substrates for continuous assay of sialyltransferase by resonance energy transfer. Anal. Biochem. 283, 39–48 (2000). 10929806 10.1006/abio.2000.4632

[b11] Carrillo L. D., Krishnamoorthy L. & Mahal L. K. A cellular FRET-based sensor for beta-O-GlcNAc, a dynamic carbohydrate modification involved in signaling. J. Am. Chem. Soc. 128, 14768–14769 (2006). 17105262 10.1021/ja065835+

[b12] Matsumoto A., Cabral H., Sato N., Kataoka K. & Miyahara Y. Assessment of tumor metastasis by the direct determination of cell-membrane sialic acid expression. Angew. Chem. Int. Ed. 49, 5494–5497 (2010). 10.1002/anie.201001220 20575125

[b13] Matsumoto A., Sato N., Kataoka K. & Miyahara Y. Noninvasive sialic acid detection at cell membrane by using phenylboronic acid modified self-assembled monolayer gold electrode. J. Am. Chem. Soc. 131, 12022–12023 (2009). 19663494 10.1021/ja902964m

[b14] Casey J. R., Grinstein S. & Orlowski J. Sensors and regulators of intracellular pH. Nat. Rev. Mol. Cell Biol. 11, 50–61 (2010). 19997129 10.1038/nrm2820

[b15] Spiro R. G. Studies on fetuin, a glycoprotein of fetal serum, I. isolation, chemical composition, and physiochemical properties. J. Biol. Chem. 235, 2860–2869 (1960). 16479679

[b16] Lichlyter D. J., Grant S. A. & Soykan O. Development of a novel FRET immunosensor technique. Biosens. Bioelectron. 19, 219–226 (2003). 14611757 10.1016/s0956-5663(03)00215-x

[b17] Reichner J. S., Whiteheart S. W. & Hart G. W. Intracellular trafficking of cell surface sialoglycoconjugates. J. Biol. Chem. 263, 16316–16326 (1988). 3182795

[b18] Oetke C. *et al.* Evidence for efficient uptake and incorporation of sialic acid by eukaryotic cells. Eur. J. Biochem. 268, 4553–4561 (2001). 11502217 10.1046/j.1432-1327.2001.02379.x

[b19] Rehman Z., Zuhorn I. S. & Hoekstra D. How cationic lipids transfer nucleic acids into cells and across cellular membranes: recent advances. J. Control. Release 166, 46–56 (2013). 23266451 10.1016/j.jconrel.2012.12.014

[b20] Rao M. & Alving C. R. Delivery of lipids and liposomal proteins to the cytoplasm and Golgi of antigen-presenting cells. Adv. Drug Delivery. Rev. 41, 171–188 (2000). 10.1016/s0169-409x(99)00064-2 10699313

[b21] Varkouhi A. K., Scholte M., Storm G. & Haisma H. J. Endosomal escape pathways for delivery of biologicals. J. Control. Release 151, 220–228 (2011). 21078351 10.1016/j.jconrel.2010.11.004

[b22] Bacon C. L., O’Driscoll E. & Regan C. M. Valproic acid suppresses G1 phase-dependent sialylation of a 65 kDa glycoprotein in the C6 glioma cell cycle. Int. J. Dev. Neurosci. 15, 777–784 (1997). 9402228 10.1016/s0736-5748(97)00019-1

[b23] Ishii S. & Volpe J. J. N-linked glycoprotein synthesis and transport during G1 are necessary for astrocytic proliferation. J. Neurosci. Res. 26, 419–427 (1990). 2122002 10.1002/jnr.490260404

[b24] Wang X., Zhang L.-H. & Ye X.-S. Recent development in the design of sialyltransferase inhibitors. Med. Res. Rev. 23, 32–47 (2003). 12424752 10.1002/med.10030

[b25] Hinderlich S., Weidemann W., Yardeni T., Horstkorte R. & Huizing M. UDP-GlcNAc 2-epimerase/ManNAc kinase (GNE): a master regulator of sialic acid synthesis in Topics in Current Chemistry (Springer, Berlin Heidelberg, 2013). 10.1007/128_2013_464 PMC4161665 23842869

[b26] Rillahan C. D., Brown S. J., Register A. C., Rosen H. & Paulson J. C. High-throughput screening for inhibitors of sialyl- and fucosyltransferases. Angew. Chem. Int. Ed. 50, 12534–12537 (2011). 10.1002/anie.201105065 PMC3245354 22095645

[b27] Green E. D., Adelt G., Baenziger J. U., Wilson S. & Van Halbeek H. The asparagine-linked oligosaccharides on bovine fetuin. Structural analysis of N-glycanase-released oligosaccharides by 500-megahertz 1H NMR spectroscopy. J. Biol. Chem. 263, 18253–18268 (1988). 2461366

[b28] Spiro R. G. & Bhoyroo V. D. Structure of the O-glycosidically linked carbohydrate units of fetuin. J. Biol. Chem. 249, 5704–5717 (1974). 4137945

[b29] Parker R. B. & Kohler J. J. Regulation of intracellular signaling by extracellular glycan remodeling. ACS Chem. Biol. 5, 35–46 (2010). 19968325 10.1021/cb9002514 PMC2825884

[b30] Otsuka H., Uchimura E., Koshino H., Okano T. & Kataoka K. Anomalous binding profile of phenylboronic acid with N-acetylneuraminic acid (Neu5Ac) in aqueous solution with varying pH. J. Am. Chem. Soc. 125, 3493–3502 (2003). 12643711 10.1021/ja021303r

[b31] Wouters F. S., Verveer P. J. & Bastiaens P. I. H. Imaging biochemistry inside cells. Trends Cell Biol. 11, 203–211 (2001). 11316609 10.1016/s0962-8924(01)01982-1

[b32] Copani A. *et al.* beta-amyloid-induced synthesis of the ganglioside Gd3 is a requisite for cell cycle reactivation and apoptosis in neurons. J. Neurosci. 22, 3963–3968 (2002). 12019315 10.1523/JNEUROSCI.22-10-03963.2002 PMC6757645

[b33] Moon S. K., Kim H. M., Lee Y. C. & Kim C. H. Disialoganglioside (GD3) synthase gene expression suppresses vascular smooth muscle cell responses via the inhibition of ERK1/2 phosphorylation, cell cycle progression, and matrix metalloproteinase-9 expression. J. Biol. Chem. 279, 33063–33070 (2004). 15175338 10.1074/jbc.M313462200

[b34] Pinho S. *et al.* Biological significance of cancer-associated sialyl-Tn antigen: modulation of malignant phenotype in gastric carcinoma cells. Cancer Lett. 249, 157–170 (2007). 16965854 10.1016/j.canlet.2006.08.010

[b35] Hashiramoto A., Mizukami H. & Yamashita T. Ganglioside GM3 promotes cell migration by regulating MAPK and c-Fos/AP-1. Oncogene 25, 3948–3955 (2006). 16491123 10.1038/sj.onc.1209416

[b36] Erikstein B. K. *et al.* Cell cycle-dependent regulation of CDw75 (β-galactoside α-2,6-sialyltransferase) on human B lymphocytes. Eur. J. Immunol. 22, 1149–1155 (1992). 1577059 10.1002/eji.1830220507

[b37] Su M. L. *et al.* Inhibition of chemokine (C-C motif) receptor 7 sialylation suppresses CCL19-stimulated proliferation, invasion and anti-anoikis. PLoS One 9, e98823 (2014). 24915301 10.1371/journal.pone.0098823 PMC4051673

[b38] Amano M. *et al.* Tumour suppressor p16(INK4a)-anoikis-favouring decrease in N/O-glycan/cell surface sialylation by down-regulation of enzymes in sialic acid biosynthesis in tandem in a pancreatic carcinoma model. FEBS J. 279, 4062–4080 (2012). 22943525 10.1111/febs.12001

[b39] Miyagi T., Takahashi K., Hata K., Shiozaki K. & Yamaguchi K. Sialidase significance for cancer progression. Glycoconj. J. 29, 567–577 (2012). 22644327 10.1007/s10719-012-9394-1

[b40] Elfeky S. A. *et al.* Diol appended quenchers for fluorescein boronic acid. Chem. –Asian J. 5, 581–588 (2010). 20127786 10.1002/asia.200900386

[b41] Lin W., Du Y., Zhu Y. & Chen X. A cis-membrane FRET-based method for protein-specific imaging of cell-surface glycans. J. Am. Chem. Soc. 136, 679–687 (2014). 24308457 10.1021/ja410086d

[b42] Haga Y. *et al.* Visualizing specific protein glycoforms by transmembrane fluorescence resonance energy transfer. Nat. Commun. 3, 907 (2012). 22713749 10.1038/ncomms1906

