# Supplementary material for: Noninvasive imaging of sialyltransferase activity in living cells by chemoselective recognition
Source: Sci Rep. 2015 Jun 5;5:10947. doi: 10.1038/srep10947 (PMC4456940; doi:10.1038/srep10947)
Supplement: Supplementary Information [file srep10947-s1.doc]

**Supplementary Information**

**Noninvasive imaging of sialyltransferase activity in living cells by chemoselective recognition**

Lei Bao^1¶^, Lin Ding^1¶^, Min Yang^2^ & Huangxian Ju^1^*****

^1^State Key Laboratory of Analytical Chemistry for Life Science, School of Chemistry and Chemical Engineering, Nanjing University, Nanjing 210093, P.R. China.

^2^Department of Pharmaceutical & Biological Chemistry, UCL School of Pharmacy, University College London, London WC1N 1AX, UK.

**^*^**Correspondence and requests for materials should be addressed to H.X.J. (email: hxju@nju.edu.cn).

^¶^These authors contributed equally to this work.

**Supplementary Methods**

**General experimental methods**

The UV-vis absorption spectra were obtained with a UV-vis spectrophotometer (Nanodrop-2000C, Nanodrop, USA). Flow cytometric analysis was performed on a Cytomics FC500 flow cytometer (Beckman-Coulter, USA). The fluorescence spectra were obtained on a RF-5301PC spectrofluorophotometer (Shimadzu, Japan). The cell images were gained on a TCS SP5 laser scanning confocal microscope (Leica, Germany). ^1^H and ^13^C spectra were measured with a Bruker Avance III 500 MHz Digital NMR spectrometer operating at 500 or 126 MHz. MALDI-TOF mass spectrometry was performed using an Applied Biosystems 4800 proteomics analyzer (Applied Biosystems) equipped with a Nd:YAG laser operating at 355 nm, a repetition rate of 200 Hz and an acceleration voltage of 20 kV. α-Cyano-4-hydroxycinnamic acid (CHCA) was used as MALDI matrix.

**Reagents**

Tetramethylrhodamine isothiocyanate (TRITC), fluorescein isothiocyanate (FITC), 3-aminophenylboronic acid (APBA), asialofetuin (AF), fetuin, bovine serum albumin (BSA), α-2,3-sialyltransferase from *Pasteurella multocida*, α-2,6-Sialyltransferase from *Photobacterium damsela*, β-galactosidase from *Escherichia coli*, cytidine-5'-monophospho-sialic acid (CMP-SA), cytidine-5'-monophosphate (CMP), uridine-5'-diphosphate (UDP), sialidase and thymidine were purchased from Sigma-Aldrich Inc. (USA). Phosphate buffer saline (PBS, 0.01 M, pH 7.4) contained 136.7 mM NaCl, 2.7 mM KCl, 8.72 mM Na_2_HPO_4_, and 1.41 mM KH_2_PO_4_. All other reagents were of analytical grade. All aqueous solutions were prepared using ultrapure water (≥ 18 MΩ, Milli-Q, Millipore).

**Cell culture and synchronization**

Human skin keratinocytes (HaCaT) and human cervical carcinoma (HeLa) cells were purchased from KeyGen Biotech (Nanjing, China), and cultured in a flask in Dulbecco’s modified Eagle’s medium (DMEM, HyClone) supplemented with 10% fetal calf serum (FCS, HyClone), penicillin (100 U mL^-1^, HyClone), and streptomycin (100 μg mL^-1^, HyClone) at 37 ^o^C in a humidified atmosphere containing 5% CO_2_. Cell number was determined using a Petroff-Hausser cell counter.

The HeLa cells were synchronized at the beginning time of S phase with a thymidine double-block method by culturing them in growth medium containing 2.5 mM thymidine for 16 h, followed in a fresh medium for 8 h and thymidine-contained medium for 16 h. After the synchronized cells were incubated in thymidine-free medium for 12 h, most cells reached the beginning point of G1 phase. The HaCaT cells were synchronized at the beginning point of G1 phase by incubation in DMEM lacking FCS for 24 h. Afterward DMEM was supplemented with 10% FCS to release the arrested cells.

**Flow cytometric analysis of synchronized HeLa cells**

After synchronized at S phase and cultured for different times, HeLa cells (1 mL, 1×10^6^ mL^-1^) were washed with PBS and centrifuged at 1000 rpm for 5 min. The cell pellet was fixed in 1 mL 75% ethanol for 30 min, centrifuged and washed with PBS to incubate with 100 μL RNase A (KeyGen Biotech) at 37 °C for 30 min. Afterward 400 μL propidium iodide staining solution (KeyGen Biotech) was added in the medium and incubated at 4 °C for 30 min in dark. With such a staining process, the DNA content could be measured on a flow cytometer through FL3 channel excited at 488 nm. At least 10000 events were analyzed for each sample. The change in DNA content gave the information of cell cycle.

**Supplementary Figures**

**
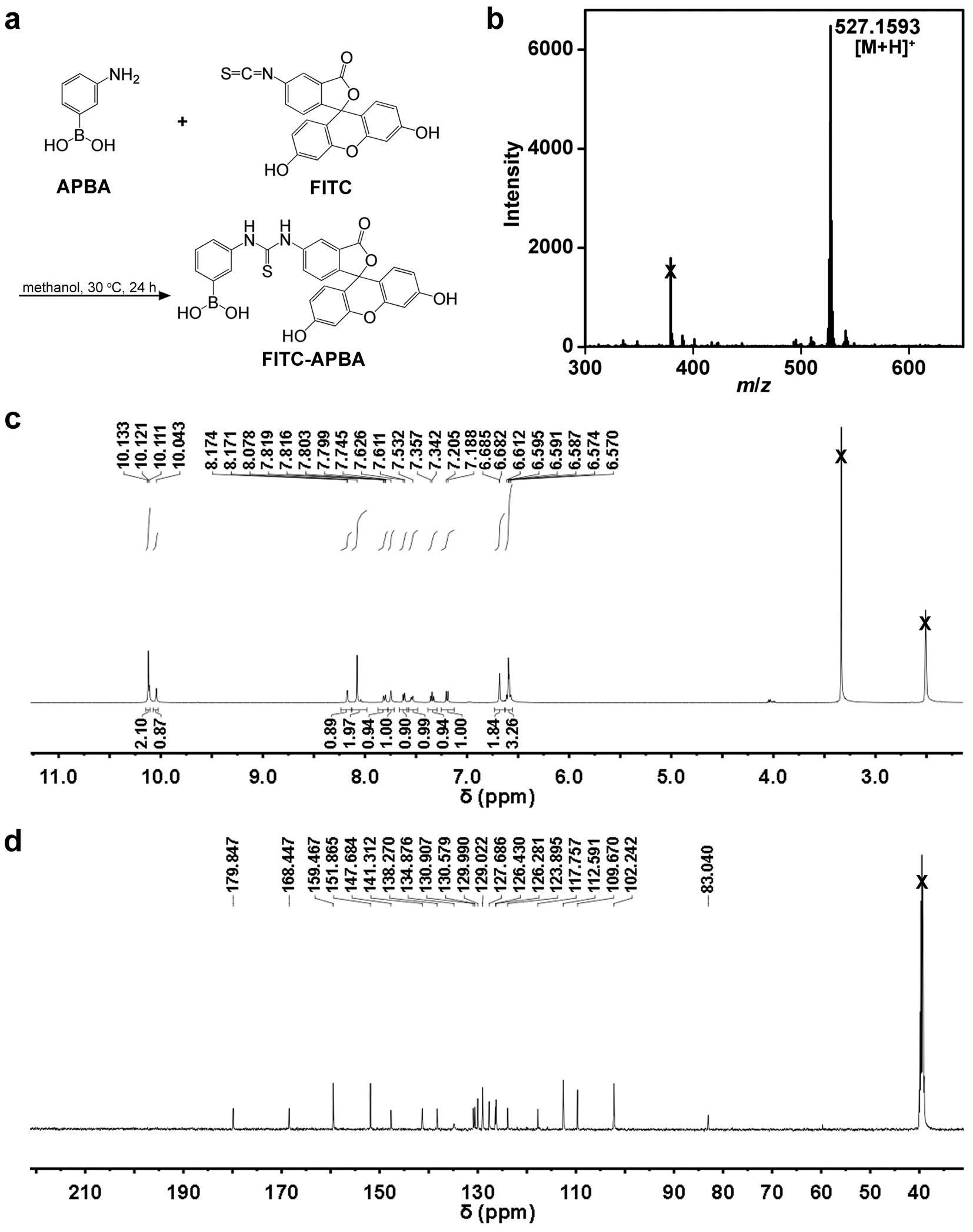
**

**Supplementary Figure S1 | Preparation and characterization of FITC-APBA.** (**a**) Synthesis of FITC-APBA. (**b**) MALDI-TOF mass spectrum. (**c**) ^1^H NMR (500 MHz, DMSO-d6) spectrum. (**d**) ^13^C NMR (126 MHz, DMSO-d6) spectrum of FITC-APBA. The matrix peak in MS and solvent peaks in NMR are indicated with “X”.


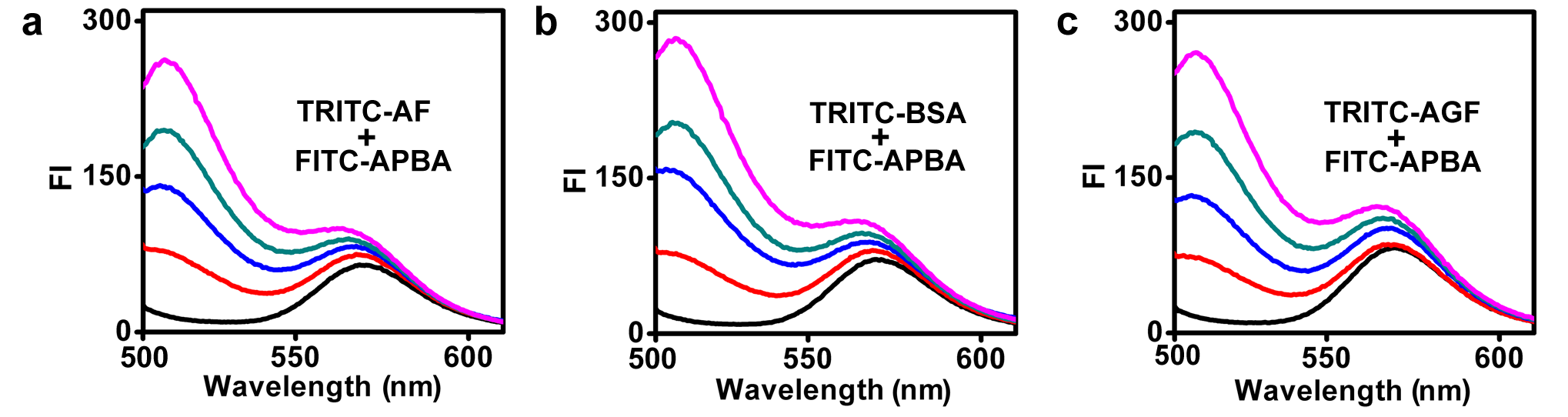


**Supplementary Figure S2 | Fluorescence intensity (FI) of FRET responses of TRITC-proteins after incubation with FITC-APBA.** (**a**) TRITC-AF, (**b**) TRITC-BSA and (**c**) TRITC-AGF at a TRITC amount of 160 nM after incubation with 0, 4, 8, 12 and 16 nM FITC-APBA (from bottom to top) for 30 min under donor excitation.


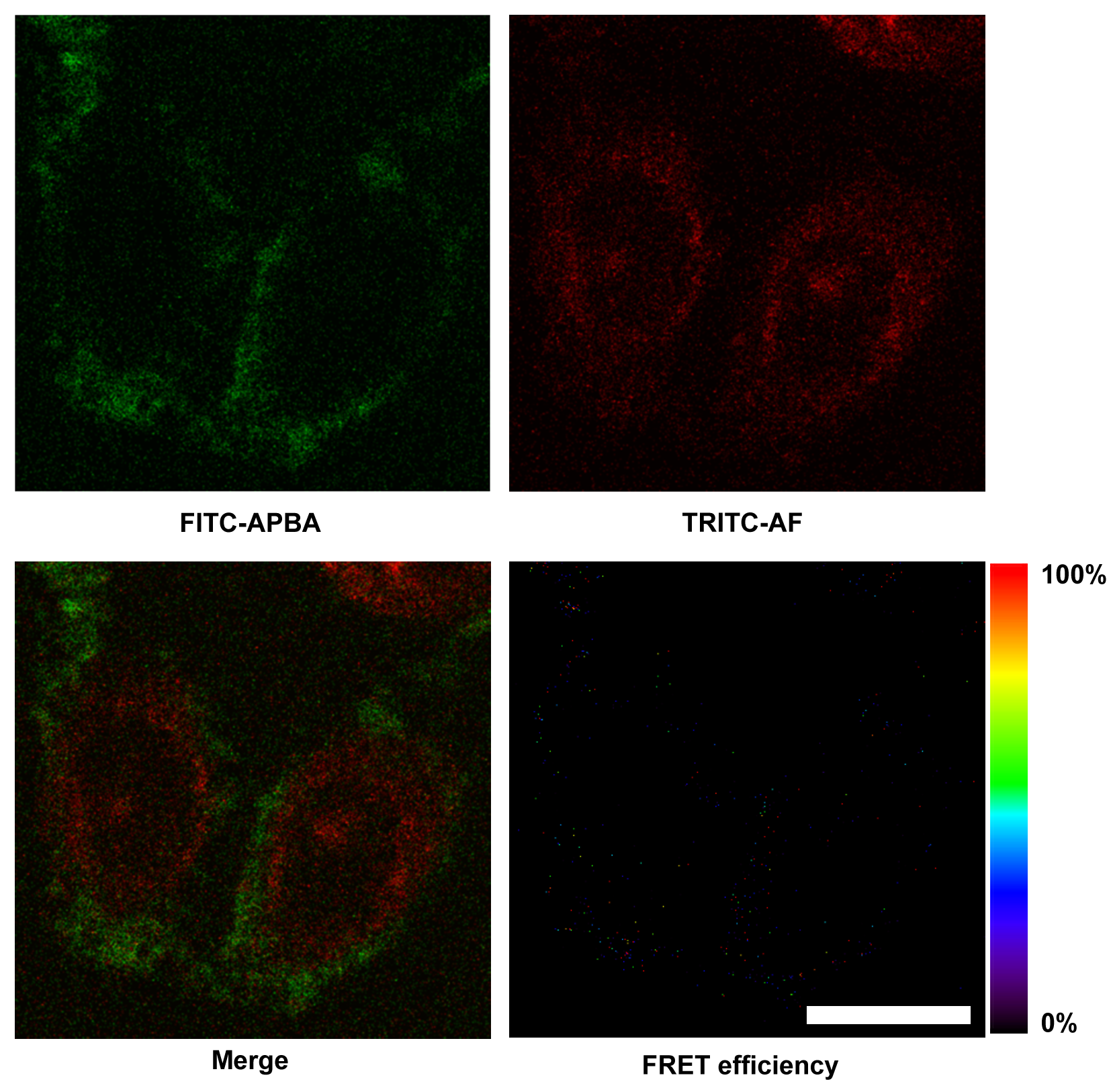


**Supplementary Figure S3 | Confocal and FRET efficiency images of G1-phase HeLa cells after incubated with FITC-APBA and TRITC-AF without liposome.** The confocal images were captured under donor and acceptor excitation, respectively. The FRET efficiency image was captured under donor excitation. Scale bar, 20 μm.


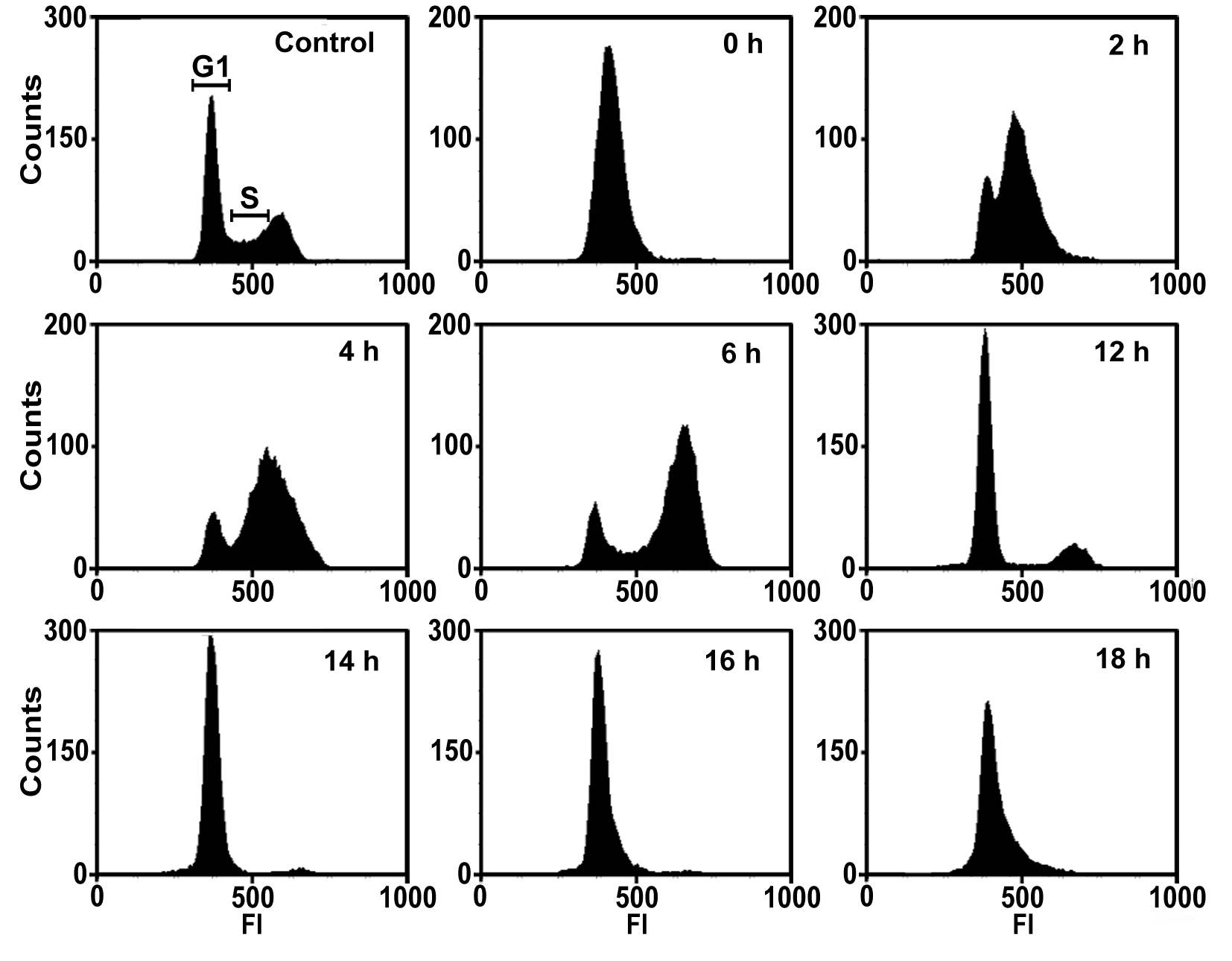


**Supplementary Figure S4 | Flow cytometric analysis of synchronized HeLa cells.** The cells are synchronized at S phase and then cultured in growth medium for different times. Most of the cells reach G1 phase at 12 h.

**
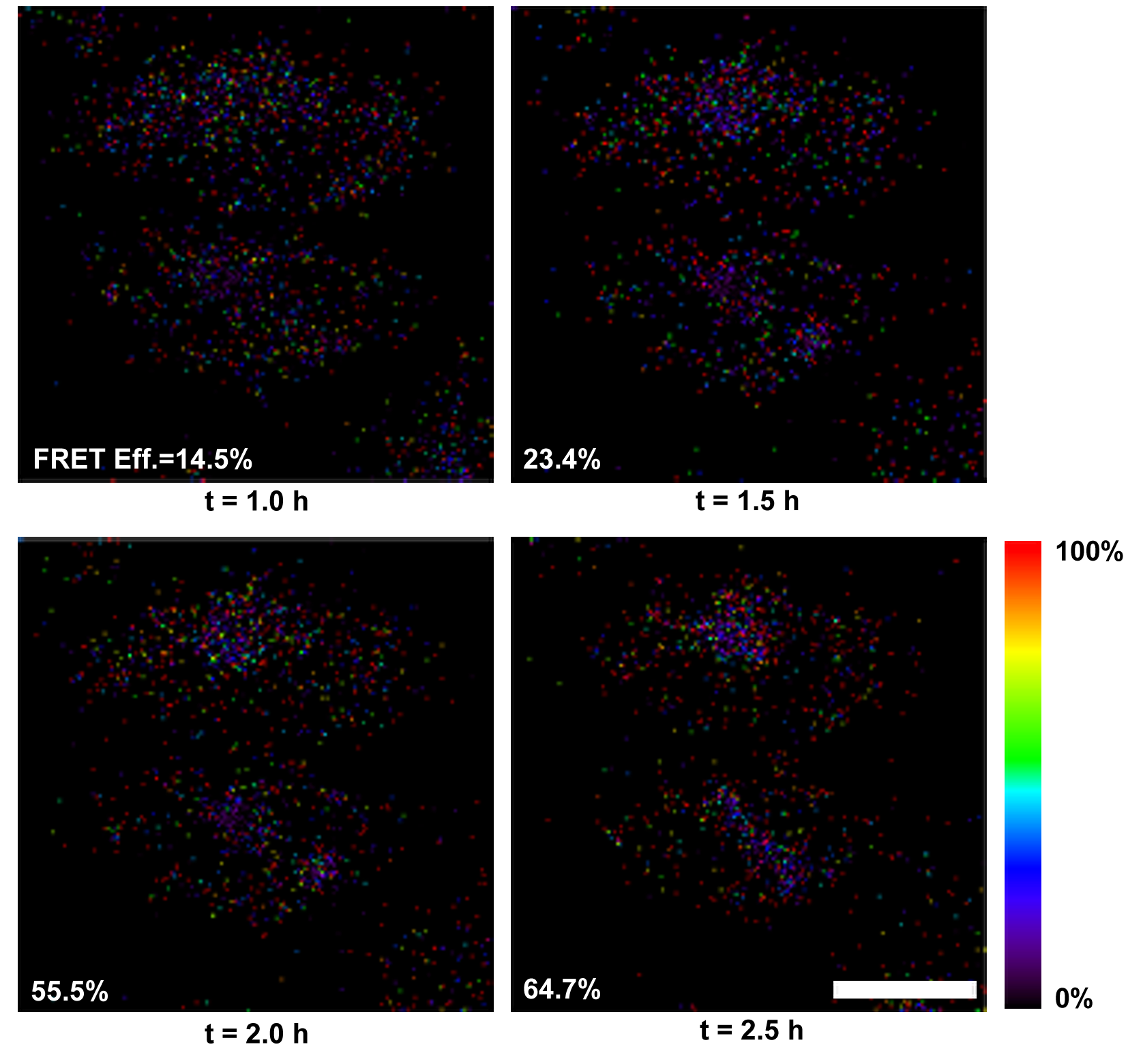
**

**Supplementary Figure S5 | Monitoring of intracellular ST activity using the sensing vesicle.** Time-course images of FRET efficiency for G1-phase HeLa cells after transfected with sensing vesicle for 2 h and then incubated in growth medium for 1 to 2.5 h. Scale bars, 20 μm.

**
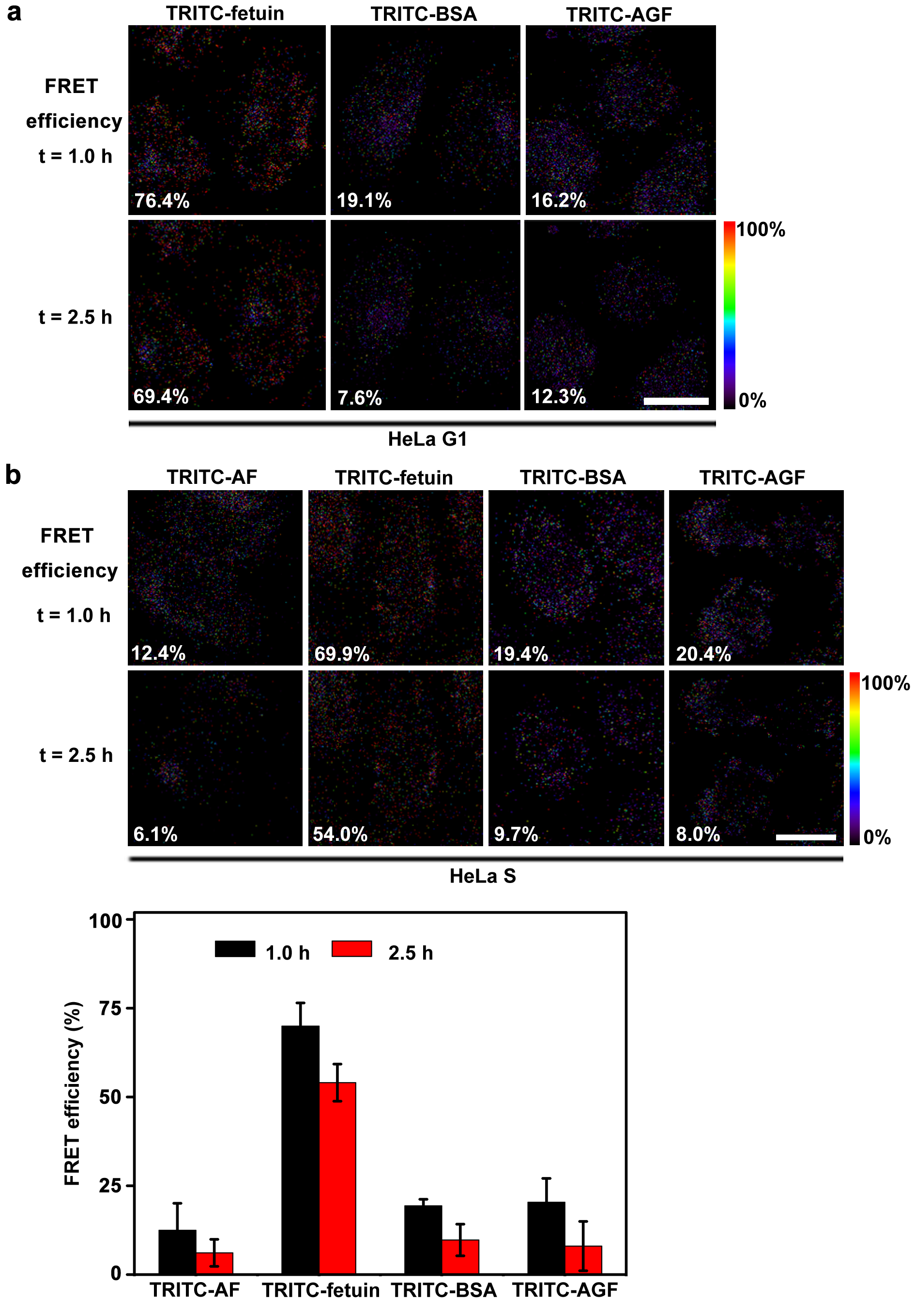
**

**
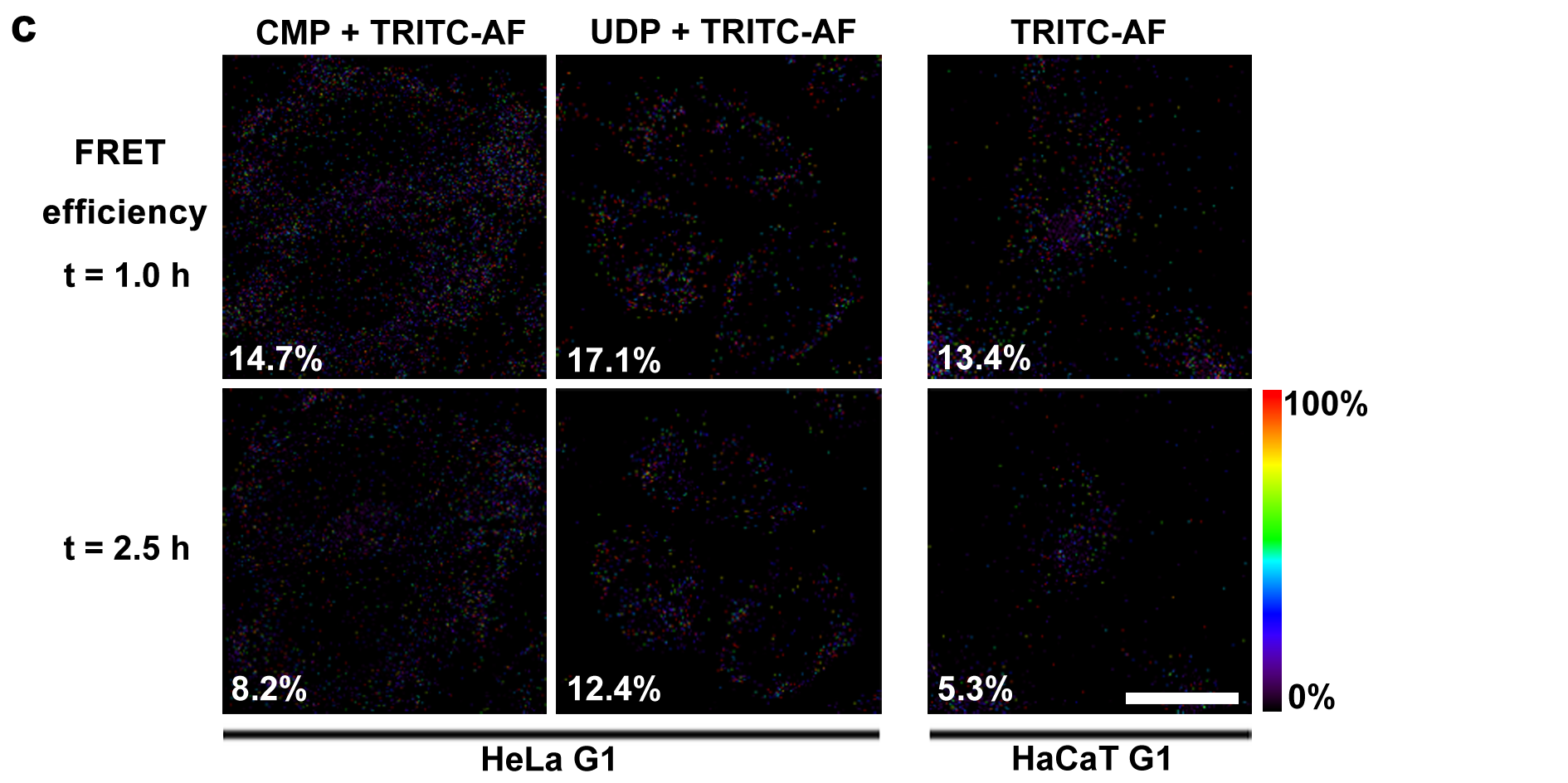
**

**Supplementary Figure S6 | Monitoring of intracellular ST activity using TRITC-proteins after transfected and then incubated in growth medium.** (**a**) FRET efficiency images of G1-phase HeLa cells. (**b**) FRET efficiency images and the corresponding quantitative data of S-phase HeLa cells. Data and error bars show the average and S.D. of three replicates. (**c**) FRET efficiency images of G1-phase HeLa cells treated with inhibitors and G1-phase HaCaT cells. Scale bars, 20 μm.


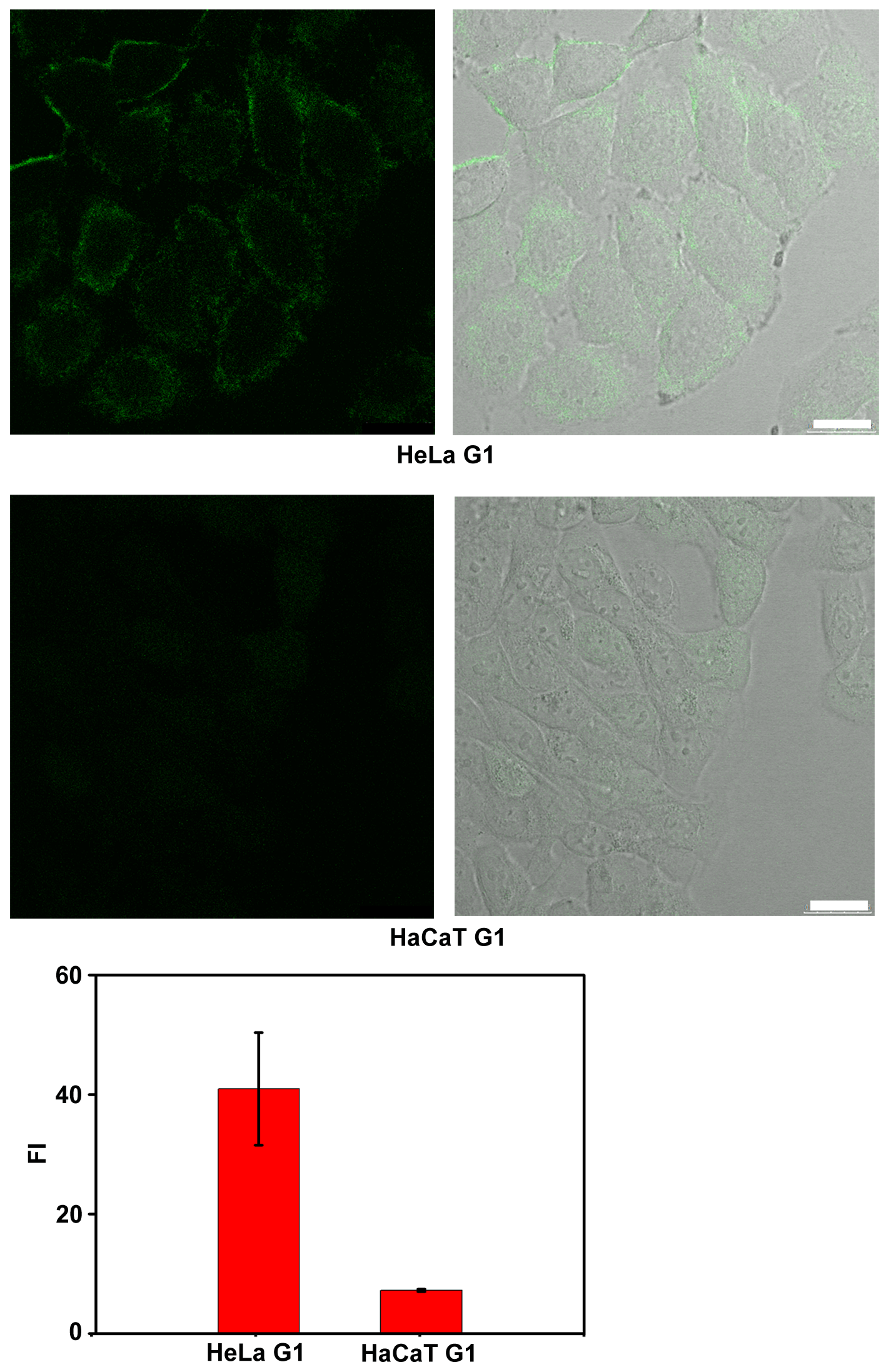


**Supplementary Figure S7 | Cell surface SA expression.** Confocal (left) and bright field merged confocal (right) images of G1-phase HeLa and HaCaT cells after incubation with FITC-APBA in PBS for 10 min. Scale bars, 20 μm. The comparison of average FI of cell membrane region was presented in bottom. Data and error bars show the average and S.D. of three replicates.
